# Anger and Cardiovascular Disease: An Old and Complicated Relationship

**DOI:** 10.5935/abc.20180176

**Published:** 2018-09

**Authors:** Carlos Eduardo Lucena Montenegro, Sergio Tavares Montenegro

**Affiliations:** 1 Pronto Socorro Cardiológico de Pernambuco (PROCAPE-UPE), Recife, PE - Brazil; 2 Centro Cardiológico Ovídio Montenegro (CECOM), Recife, PE - Brazil; 3 Real Hospital de Beneficência Portuguesa de Pernambuco, Recife, PE - Brazil

**Keywords:** Anger/physiology, Stress,Psychological, Depression, Coronary Artery Disease, Anxiety Disorders

Negative feelings have long been related to health problems. Buddhism, for example, refers to anger as one of the three "poisons of the mind" (greed, anger and madness).^1^ In the first edition of *Circulation*, there was already an article suggesting the association between stress and cardiovascular disease.^2^

In the last decades, several studies have tried to correlate psychosocial factors, such as anger, anxiety, depression and stress, with coronary artery disease (CAD), demonstrating the increase in the incidence of CAD in patients with a higher incidence of these psychic conditions, for example, with a marked increase in events of acute myocardial infarction (AMI) between 2008 and 2009, in the United States, when there was a stock market crash.^3-5^ This relationship tends to be significantly more important in women, since factors such as low socioeconomic status and double working hours (conciliation of employment with maternity), among other factors, are more common in the female population.^5^ More recently, a longitudinal study of cohort was able to demonstrate the association between the activity of the cerebral amygdala (area involved with the emotions) and the increased risk of cardiovascular events.^6^

An important problem in studies that attempt to objectively demonstrate the relationship between anger and CAD is the difficulty of objectively measuring emotions, including anger. The study published in this edition of the Brazilian Articles of Cardiology by Schmidt et al.^7^ measures the female patients' anger analyzed through the State-Trait Anger Expression Inventory of Spielberger (STAXI).^7^ This score is validated in Brazil for the analysis of anger, being even recommended by the Federal Council of Psychology, and certainly underused in our settings.^8^

Dimsdale, in 2008, in his brilliant work on the state of the art of the relationship between psychological stress and cardiovascular disease explains that "anyone who starts reading the papers that analyze this association immediately notes that part of the problem is that the term stress is used in a variety of ways," so that any student who wants to deepen into this area has to be careful about the nuances that involve this subject.^9^

The study by Schmidt et al.^10^ also shows that, as important as demonstrating the value of anger as a risk factor for the presence of CAD, it is evident that anger management may also play a role, as the study shows that women who have shown less control of anger had a tendency to the presence of CAD on the coronary angiography. However, this issue remains controversial. A systematic review of 36 studies, including 12,841 patients, of which 18 trials evaluated anger control, showed that there is no decrease in cardiovascular death, or need for new revascularizations, when psychotherapeutic strategies are implemented for patients with anger, anxiety or depression. There was a trend towards a decrease in nonfatal AMI in the intervention group, but the 2 largest trials involved in this review were null for this outcome.^11^

In summary, we still need more and better evidence to assess whether this millenarian relationship between anger and CAD is a modifiable risk factor or not, and whether we can intervene in these patients. Schmidt's study has great value because it is one of the few that have made gender differentiation, which is very important in the analysis of the risk factors for CAD. In addition, the fact that it involves only women makes it more valuable, since it is a population that is often "forgotten" in prospective studies. In addition, prospective analyzes in this regard are always welcome to improve the quality of the data we have so far on this very relevant topic.
